# Rice Bran Metabolome Contains Amino Acids, Vitamins & Cofactors, and Phytochemicals with Medicinal and Nutritional Properties

**DOI:** 10.1186/s12284-017-0157-2

**Published:** 2017-06-02

**Authors:** Iman Zarei, Dustin G. Brown, Nora Jean Nealon, Elizabeth P. Ryan

**Affiliations:** 1 Department of Environmental & Radiological Health Sciences, College of Veterinary Medicine and Biological Sciences, Colorado State University, 1680 Campus Delivery, Fort Collins, CO 80523 USA; 2 Institute of Human Nutrition and Food, College of Human Ecology, University of the Philippines Los Baños, Los Baños, 4031 Laguna Philippines

**Keywords:** Rice bran, Functional food, Metabolomics, Medicinal properties, Chronic diseases, Phytochemicals, Infectious diseases

## Abstract

**Background:**

Rice bran is a functional food that has shown protection against major chronic diseases (e.g. obesity, diabetes, cardiovascular disease and cancer) in animals and humans, and these health effects have been associated with the presence of bioactive phytochemicals. Food metabolomics uses multiple chromatography and mass spectrometry platforms to detect and identify a diverse range of small molecules with high sensitivity and precision, and has not been completed for rice bran.

**Results:**

This study utilized global, non-targeted metabolomics to identify small molecules in rice bran, and conducted a comprehensive search of peer-reviewed literature to determine bioactive compounds. Three U.S. rice varieties (Calrose, Dixiebelle, and Neptune), that have been used for human dietary intervention trials, were assessed herein for bioactive compounds that have disease control and prevention properties. The profiling of rice bran by ultra-performance liquid chromatography-tandem mass spectrometry (UPLC-MS/MS) and gas chromatography–mass spectrometry (GC–MS) identified 453 distinct phytochemicals, 209 of which were classified as amino acids, cofactors & vitamins, and secondary metabolites, and were further assessed for bioactivity. A scientific literature search revealed 65 compounds with health properties, 16 of which had not been previously identified in rice bran. This suite of amino acids, cofactors & vitamins, and secondary metabolites comprised 46% of the identified rice bran metabolome, which substantially enhanced our knowledge of health-promoting rice bran compounds provided during dietary supplementation.

**Conclusion:**

Rice bran metabolite profiling revealed a suite of biochemical molecules that can be further investigated and exploited for multiple nutritional therapies and medical food applications. These bioactive compounds may also be biomarkers of dietary rice bran intake. The medicinal compounds associated with rice bran can function as a network across metabolic pathways and this metabolite network may occur via additive and synergistic effects between compounds in the food matrix.

**Electronic supplementary material:**

The online version of this article (doi:10.1186/s12284-017-0157-2) contains supplementary material, which is available to authorized users.

## Background

Rice (*Oryza sativa* L.) is an essential staple food for more than half of the world’s population (Hu et al. 2014; Qian et al. 2016) and is grown in more than 100 countries worldwide (Muthayya et al. 2014). Rice bran, the outer covering of the rice grain, contains a unique profile of phytochemicals with medicinal and nutritional properties that are beneficial to human health, some of which have been targeted for nutraceutical development for cancer (Henderson et al. 2012; Verschoyle et al. 2007), type 2 diabetes (Cheng et al. 2010; de Munter et al. 2007; Qureshi et al. 2002), lipid metabolism regulation (Kuriyan et al. 2005; Qureshi et al. 1997; Shibata et al. 2016; Wang et al. 2015), immune regulatory processes (Wang et al. 2015), and obesity (Ham et al. 2015). Furthermore, we recently showed whole rice bran can protect against enteric pathogens such as *Salmonella enterica serovar* Typhimurium, human rotavirus, and human norovirus (Goodyear et al. 2015; Kumar et al. 2012; Lei et al. 2016; Yang et al. 2015). Rice bran contains non-saponifiable lipids (i.e. gamma oryzanol), vitamin E (e.g. tocopherols and tocotriols), polyphenols (e.g. ferulic acid caffeic acid and salicylic acid), and phytosterols (e.g. beta-sitosterol) (Henderson et al. 2012) with reported health properties. Many of these compounds are available in the lipid fraction and also known as rice bran oil (Charoonratana et al. 2015; Iqbal et al. 2003; V. Panala, 2009). Additional compounds from other chemical classes in rice bran merit attention and can be identified via high throughput techniques, such as global, non-targeted metabolomics that can assess a large profile of small molecules present in the whole food. Given the emphasis in previous studies on rice bran lipids (Forster et al. 2013), this analysis focused on rice bran amino acids, cofactors & vitamins, and secondary metabolites that have medicinal and nutritional properties important to human health.

Despite the large body of scientific evidence on rice bran bioactivity, rice bran remains underutilized in human health and nutrition because it is considered an animal feed and is known to undergo hydrolytic rancidity after processing from whole grain rice (da Silva et al. 2006; Ramezanzadeh et al. 1999a, b). Thermal treatments applied to rice bran have helped to stabilize it and prevents rancidity by the inactivation of lipases and peroxidases. One major obstacle to achieving widespread human consumption and acceptance of rice bran is the global perception that rice bran is an animal feed (Ramezanzadeh et al. 1999a).

Food metabolomics, or “Foodomics”, provides information on the presence and relative abundance of all compounds in a food matrix. Food metabolome studies have shown compounds across diverse chemical classes such as amino acids, lipids, sugars, peptides, organic acids, phenolic compounds and other secondary metabolites (Wishart, 2008). Entire metabolite profiles have been completed on several foods including cooked and uncooked rice grain (Heuberger et al. 2010; Hu et al. 2014; Kim et al. 2013a, 2013b), grape (*Vitis vinifera* L.) (Luca Narduzzi, 2015), human milk (Andreas et al. 2015; Wu et al. 2016), tomato (Moco et al. 2006), citrus juice (Arbona et al. 2015), and several other foods and crops (e.g., carrot, beer, wine, and coffee) (Johanningsmeier et al. 2016) through non-targeted screening methods. Nutritional metabolomics is an experimental approach that uses small molecule profiling to integrate the effects of diet on nutrition, and thus can be used to evaluate the health effects of foods at an individual level (Jones et al. 2012). Integrating food and nutritional metabolomic approaches can increase our knowledge on the bioactivity of food metabolites, and may increase evidence for metabolic mechanisms by which foods elicit important health effects (Capozzi and Bordoni, 2013; Herrero et al. 2012). Accurate food metabolite profiles in regards to food and nutritional metabolomics may also assist in the quantification of dietary intakes and specific food biomarkers.

The goal of the food metabolome approach applied herein was to obtain a complete characterization of the rice bran small molecule profile for bioactive components. This study used non-targeted metabolomics to investigate heat-stabilized rice bran from three U.S. rice cultivars for the identification of metabolites with medicinal and nutritional properties. These varieties were chosen for profiling based on human consumption in clinical trials, whereby rice bran intake improved intestinal health parameters by modulating gastrointestinal microbiota and host immunity (Borresen et al. 2016; Sheflin et al. 2016; Yang et al. 2015). The hypothesis was that rice bran contains a distinct stoichiometry of small molecules, covering multiple classes of phytochemicals, including but not limited to amino acids, cofactors & vitamins, and secondary metabolites that have medicinal properties and contribute to the nutritional benefits of rice bran as a whole food. A thorough examination of metabolites across chemical classes revealed a complex network of metabolic pathways that have not been previously examined for rice bran. A detailed analysis of rice bran functional food components allowed for a thorough understanding of how a suite of metabolites in a single food can exhibit therapeutic and preventive medicine properties.

## Results

### Non-Targeted Rice Bran Metabolomics

The metabolite profile composition of rice bran revealed 453 metabolites with known identity that were clustered into the following metabolic pathways: amino acid (126 metabolites), carbohydrate (35 metabolites), cofactors & vitamins (28 metabolites), energy (11 metabolites), lipids (137 metabolites), nucleotides (40 metabolites), peptides (28 metabolites), secondary metabolites (55 metabolites), and xenobiotics (8 metabolites). Total metabolite numbers for each metabolic pathway are shown in Additional file 1: Table S1. Approximately 46% of total identified metabolites (209 metabolites) were classified as amino acids, cofactors & vitamins, and secondary metabolites and were interrogated for their potential human health-promoting properties. Medicinal and health promoting attributes were previously reported in the scientific literature for 65 rice bran metabolites from these three pathways. Table 1 lists the 29 amino acids, Table 2 lists the 13 cofactors & vitamins, and Table 3 lists the 23 secondary metabolites with previously reported health beneficial properties. A total of 16 out of 65 metabolites (noted by ^1^ in tables) with medicinal/nutritional properties were identified from this metabolome analysis that had not previously been reported from rice bran. These included 9 amino acids, 2 cofactors & vitamins, and 6 secondary metabolites.

**Table 1:** Median-scaled relative abundance of rice bran amino acids metabolites with medicinal properties

Metabolite	HMDB ID	Calrose	Dixiebelle	Neptune	Function	References
4-guanidinobutanoate (Rhapontigenin)^a^	03464	99.84	194.48	153.27	• Anti-hyperlipidemic • Antifungal (*Candida albicans* in vitro) • Antioxidant (protection against cellular DNA damage caused by intracellular reactive oxygen species (ROS))	(Jo et al. 2014) (Kim et al. 2013a, b) (Zhang et al. 2007)
4-hydroxycinnamic acid	02035	3.61	6.62	9.49	• Antioxidant (protective against oxidative damage caused by ROS)	(Shang et al. 2015)
5-oxoproline (Pyroglutamic acid)	00267	10.80	22.81	41.36	• Increases the release of acetylcholine (Ach) and GABA from the cortical surface to improve the age associated memory impairment	(Antonelli et al. 1984) (Grioli et al. 1990)
3-(4-hydroxyphenyl) lactate	00755	0.35	0.57	1.09	• Antioxidant (decrease the ROS production in neutrophils and mitochondria) • Antifungal (against main genera: *Penicillum, Aspergillus* and *Fusarium*)	(Beloborodova et al. 2012) (Dallagnol et al. 2011)
α-hydroxyisocaproic acid (Leucic acid)^a^	00746	0.16	0.21	0.45	• Antifungal (against *Candida* and *Aspergillus* species) • Antibacterial (broad spectrum bacteriostatic properties) • Anti-catabolic (inhibitory effect on various matrix metalloproteinase enzymes, which are responsible for degradation of various connective and protein tissues → muscle gaining)	(Sakko et al. 2014) (Sakko et al. 2012) (Mero et al. 2010)
Agmatine^a^	01432	6.89	0.95	5.23	• Anti-nociceptive (without affecting morphine-induced gastrointestinal transit) • Anti-hypertensive • Cancer chemopreventive • Renal sodium regulation • Antihyperglycemic (increases insulin release from rat pancreatic islets of Langerhans cells)	(Raasch et al. 2001) (Piletz et al. 2013)
Betaine	00043	249.03	197.66	308.76	• Protects against atherosclerosis (helps reduce higher levels of homocysteine) • Prevents hepatic steatosis, prevents cirrhosis (prevents or reduces accumulation of fat in the liver) • Protects against genetic instability, senescence, and cancer through DNA methylation	(Craig, 2004)
Citrulline	00904	2.72	0.34	ND	• Anti-hypertensive (increases the arginine availability as its precursor and arginine sustains increase in nitrogen oxide (NO) production) • Anti-hyperlipidemic	(Kaore et al. 2013)
Cystathionine	00099	ND	0.75	1.05	• Anti-inflammatory	(Zhu et al. 2015)
Carboxyethyl-GABA	02201	11.50	12.42	16.56	• Immune modulator	(Cerino et al. 1988)
GABA (Gamma-aminobutyric acid)	00112	163.45	121.42	102.60	• Inhibitory neurotransmitter → Relaxant, anxiolytic and anti-convulsive (antiepileptic)	(Foster and Kemp, 2006) (Chapouthier and Venault, 2001)
Gentisate^a^	00152	ND	0.07	0.27	• Anti-inflammatory (via free radical scavenging) • Antirheumatic	(Carlin et al. 1985) (Clarke and Mosher, 1953; Kleinsorge and Pohl, 1953)
Glutamate	00148	777.00	581.99	571.80	• Required for central nervous system function and treating neuropsychological conditions • Immunomodulator (development of T-cell-mediated immunity by stimulating glutamate-specific receptors)	(Hettema et al. 2006) (Pacheco et al. 2007) (Pietersen et al. 1998)
Glutathione, reduced (GSH)	00125	25.76	21.87	14.02	• Antioxidant (as a nucleophile and a reductant, and can react with electrophilic or oxidizing species	(Pompella et al. 2003)
Hydrocinnamic acid	00764	0.07	0.09	ND	• Anti-inflammatory (control of the degranulation of mast cells, basophils and neutrophils)	(Panico et al. 2005)
Indoleacetate^a^	00197	1.85	1.73	0.98	• Cancer chemopreventive	(Folkes and Wardman, 2001)
N-Acetyl histidine (NAH)	32055	8.65	11.33	3.36	• Anti-cataract formation (it is one the two major constituents of the vertebrate brain and eye)	(Baslow, 1998; Baslow and Guilfoyle, 2015)
Serotonin	00259	4.71	2.50	14.11	• Neurotransmitter • Antiemetic	(Peroutka et al. 1981) (De-Miguel and Trueta, 2005)
N-Acetylserotonin	01238	ND	0.09	0.41	• Anti-insomnia (this is the immediate precursor of melatonin) • antidepressant & anxiolytic • Anti-hypertensive • Antioxidant (lowers resting levels of ROS in peripheral blood lymphocytes, and inhibits nitric oxide synthase) • Anti-inflammatory	(Touitou, 2001) (Oxenkrug, 1999; Oxenkrug et al. 2007) (Reiter et al. 1999) (Perianayagam et al. 2005)
N-acetylleucine^a^	11756	0.12	0.17	0.41	• Vertigo treatment	(Kanchan Rao Singh, 2012; Przybylski, 2008)
N-Acetyl-L-tyrosine	00866	0.10	0.20	0.44	• Treatment of neurotransmitter dysfunction (administration of this amino acid acts as a precursor of catecholamine, dopamine and serotonin)	(M. Hinz, 2003; M. C. Hinz, 2009)
N-acetyltryptophan	13713	ND	0.19	0.55	• Antioxidant (diminishes oxidation of human serum albumin) • Neuro-protective (Treatment of neurodegenerative disease such as amyotrophic lateral sclerosis)	(Anraku et al. 2004) (W. Li et al. 2015)
N-methyltyrosine (Metyrosine)	14903	0.06	ND	ND	• Anti-hypertensive (inhibits tyrosine hydroxylase)	(DrugBank; Scriabine et al. 1978)
Norvaline	13716	0.13	ND	0.07	• Anti-inflammatory (via inhibition of ribosomal protein S6 kinase beta-1 (S6K1)) • Improves sperm motility, count and viability in diabetic rats (inhibits the arginase enzyme and increases arginine availability as substrate to interact with endothelial nitric oxide synthase (eNOS))	(Ming et al. 2009) (De et al. 2016)
Ornithine	03374	1.42	0.12	0.27	• Antifatigue (increases release of human growth hormone by stimulating pituitary gland)	(Sugino et al. 2008)
Phenyllactic acid^a^	00779	0.28	0.37	0.59	• Antifungal (against *Fusarium graminearum* IDM623, *Endomyces fibuliger* IDM3812, *Penicillium expansum IDM/FS2*, *Aspergillus niger* IDM1, and *Monilia sitophila* IDM/FS5) • Antibacterial (against *Klebsiella oxytoca* (g-), *Providencia stuartii* (g-), *Enterococcus faecalis* (g+), *Staphylococcus aureu* (g+) *and Listeria monocytogenes* (g+))	(Lavermicocca et al. 2000) (Dieuleveux et al. 1998)
Picolinic acid	02243	0.22	0.66	0.19	• Increases the bioavailability of elements such as zinc, iron, copper, manganese and molybdenum in the human body • Anti-inflammatory	(Grant et al. 2009) (Bosco et al. 2000)
Taurine^a^	00251	1.41	1.92	1.92	• Antioxidant (found in large quantities in the neutrophil and excitable tissues, is a powerful scavenger of hypochlorous acid) • Lowers lead and cadmium levels in blood and tissues • Obesity prevention (increases energy metabolism in white adipose tissue) • Anti-hypertensive • Neuroprotective against glutamate excitotoxicity • Anti-hyperlipidemic	(Christophersen, 2012) (Gurer et al. 2001) (Sinha et al. 2008) (Tsuboyama-Kasaoka et al. 2006) (Leon et al. 2009) (El Idrissi et al. 2003) (Yanagita et al. 2008) (Huxtable, 1992)
Trans-urocanate (t-Urocanic acid)	00301	0.58	0.77	2.24	• Ultraviolet (UV) protectant (natural sunscreen)	(Egawa et al. 2010)

*ND* Not Detected

^a^Newly identified for expression in rice bran

**Table 2:** Median-scaled relative abundance of rice bran cofactors & vitamins metabolites with medicinal properties

Metabolite	HMDB ID	Calrose	Dixiebelle	Neptune	Function	References
Alpha-tocopherol	01893	0.02	0.05	0.03	• Antioxidant (antioxidant activity against (Fe^2+^ + ascorbate) and (Fe ^2+^ + NADPH)-induced lipid peroxidation, decreases plasma and low-density lipoprotein (LDL) oxidizability) • Anti-hypertensive (inhibits smooth muscle cell proliferation by inhibiting protein kinase C activity)	(Serbinova et al. 1991) (Kontush et al. 1996) (Chatelain et al. 1993)
Alpha-tocopherol acetate	34227	0.02	0.04	ND	• Boosts alpha-tocopherol antioxidant bioactivity	(Brigelius-Flohe and Traber, 1999)
Alpha-tocotrienol	06327	0.01	0.08	0.05	• Antioxidant	(Serbinova et al. 1991)
Beta-tocopherol	06335	0.001	ND	ND	• Antioxidant (free radical scavenging)	(Kadoma et al. 2006)
Delta-tocopherol	02902	ND	0.01	ND	• Antioxidant (free radical scavenging) • Anti-hypertensive (inhibits smooth muscle cell proliferation by inhibiting protein kinase C activity)	(Kadoma et al. 2006) (Chatelain et al. 1993)
Gamma-tocopherol	01492	ND	0.07	0.03	• Anti-inflammatory • Anti-hypertensive (inhibits smooth muscle cell proliferation by inhibiting protein kinase C activity)	(Jiang et al. 2000) (Chatelain et al. 1993)
Gamma-tocotrienol	12958	0.03	0.17	0.12	• Radio-protector • Anti-hypertensive	(Ghosh et al. 2009)
Glucarate (saccharate)^a^	00663	0.89	0.13	ND	• Cancer chemopreventive (by inhibiting serum β-glucuronidase)	(Lampe et al. 2002) (Hanausek et al. 2003)
Nicotinamide (vitamin B3)	01406	2.00	2.04	4.28	• Anti-inflammatory (treatment of skin disorders such as acne vulgaris) • Cancer chemopreventive (treatment of non-melanoma skin cancer) • Treatment of Alzheimer's disease (by inhibiting of poly(ADP-ribose) polymerase-1 (PARP-1) and neuro-inflammatory enzyme)	(Khodaeiani et al. 2013; Niren, 2006) (A. C. Chen et al. 2015; Surjana et al. 2012) (Turunc Bayrakdar et al. 2014)
Nicotinate	01488	12.11	12.11	21.17	• Anti-hyperlipidemic	(Duggal et al. 2010; Figueroa et al. 2015; Zema, 2000)
Pyridoxine (Vitamin B6)	02075	2.67	4.22	5.03	• Required for biosynthesis of amino acids, sugars and fatty acids, and major centrally-acting neurotransmitters (e.g. serotonin, dopamine, and GABA) • Anti-hypertensive • Anti-hyperlipidemic • Anti-hyperglycemic • Cancer chemopreventive (colon cancer)	(Percudani and Peracchi, 2009) (Yarlagadda and Clayton, 2007) (Hellmann and Mooney, 2010) (Larsson et al. 2010)
Threonic acid^a^	00943	36.15	20.99	16.44	• Prevention of androgen-driven balding • Increases bioavailability of vitamin C for T-cells (calcium L-threonate) • Increases bioavailability of iron (is used for treatment of iron deficiency anemia	(Kwack et al. 2010) (Fay and Verlangieri, 1991) (X. X. Li et al. 2005)
Trigonelline	00875	608.11	0.77	11.59	• Cancer chemopreventive	(Hirakawa et al. 2005) (Liao et al. 2015)

ND: Not Detected

^a^Newly identified for expression in rice bran

**Table 3:** Median-scaled relative abundance of rice bran secondary metabolites with medicinal properties

Metabolite	HMDB ID	Calrose	Dixiebelle	Neptune	Function	References
4-hydroxybenzoate^a^	00500	1.18	1.87	1.04	• Antimicrobial (broad spectrum bacteriostatic and fungistatic properties • Antioxidant (scavenges free radicals to reduce skin damage)	(Kosova et al. 2015) (Barreca et al. 2016)
Abscisate	35140	0.62	0.31	0.40	• Anti-inflammatory (type II diabetes and obesity-related inflammation)	(Guri et al. 2007)
Alpha-amyrin^a^	36657	0.06	0.08	0.12	• Anti-inflammatory • Antimicrobial (broad spectrum bacteriostatic properties)	(Liliana Hernández Vázquez, 2012)
Apigenin	02124	ND	0.08	0.46	• Cancer chemopreventive • Anti-inflammatory and anti-metastatic agent	(Balasubramanian et al. 2006) (Vargo et al. 2006)
Astragalin	37429	ND	ND	2.89	• Anti-histaminergic (reduces IgE production) • Anti-parasitic (against *Fasciolopsis buski*) • Anti-inflammatory (reduces inflammation through nuclear factor NF-kB inhibition) • Neuroprotective (protects neurons from hydrogen peroxide-mediated cell death during neuroblastoma)	(Kotani et al. 2000) (Ananta Swargiary 2015) (Chung et al. 2016)
Benzoate	01870	4.38	3.50	3.36	• Antimicrobial (broad spectrum bacteriostatic and fungistatic properties)	(Nascimento et al. 2000)
Caffeate	01964	ND	0.20	1.23	• Antioxidant • Cancer chemopreventive (functions as an antiproliferative chemotherapeutic agent)	(Olthof et al. 2001) (Rajendra Prasad et al. 2011)
Chlorogenic acid	03164	ND	ND	0.03	• Antioxidant • Anti-diabetic (influences glucose metabolism by inhibiting alpha-glucosidase) • Anti-septic arthritis caused by *Candida albicans*. • Sleep-wake cycle modulator	(Olthof et al. 2001) (Upadhyay and Mohan Rao, 2013)
Chrysoeriol	30667	0.49	0.16	0.92	• Antioxidant • Anti-inflammatory(Inhibits lipid peroxidation, inhibits production of superoxide anion by xanthine/xanthine oxidase system, inhibits the lipopolysaccharideinduction of the nitric oxide synthase (iNOS) gene.)	(Mishra et al. 2003) (Choi et al. 2005)
Cinnamate	00930	0.31	0.12	0.18	• Antimicrobial (anti-mycobacterial agent that can be developed against tuberculosis) • Cancer chemopreventive (anti-proliferative activity against melanoma cells and lung carcinoma cells, inhibition of histone deacetylases in colon cancer cells) • Anti-obesogenic • Anti-hypertensive (inhibits serum lipase and angiotensin-converting enzyme) • Cardio-protective (In electrocardiography, it decreases the ST segment elevation induced by acute myocardial ischemia) • Antioxidant • Anti-inflammatory	(Y. L. Chen et al. 2011) (Zhu et al. 2016) (Mnafgui et al. 2015) (Song et al. 2013)
Ergothioneine^a^	03045	ND	0.79	0.99	• Antioxidant	(Aruoma et al. 2012)
Ferulate	00954	6.22	10.28	19.69	• Antioxidant	(Kanski et al. 2002 2002)
Indolin-2-one	-	1.19	0.30	3.80	• Cancer chemopreventive (anti-proliferative and inhibits thioredoxin reductase (TrxR))	(Kaminska et al. 2016)
Luteolin	05800	ND	0.04	1.67	• Antioxidant (scavenges ROS) • Antimicrobial (broad spectrum gram positive bacteriostatic properties and yeast)	(Lin et al. 2008) (Singh et al. 2016)
Piperidine^a^	34301	0.76	0.69	0.91	• Anti-hypertensive	(Aisaka et al. 1985; Patel et al. 2006)
Quinate^a^	03072	8.86	1.65	7.27	• Anti-inflammatory • Antioxidant	(Pero et al. 2009)
Salicylate	01895	6.67	0.48	2.12	• Anti-diabetic (reduces blood glucose via activation of adenosine monophosphate-activated protein kinase) • Anti-inflammatory	(Hawley et al. 2012)
Sinapic acid	32616	0.15	1.99	2.22	• Anti-inflammatory • Anti-diabetic (increases expression of glucose transporter type 4)	(Yun et al. 2008) (Cherng et al. 2013)
Sitostanol (Stigmastanol)	00494	ND	0.10	0.06	• Anti-hyperlipidemic	(Batta et al. 2006)
Syringic acid	02085	0.32	0.36	0.49	• Antioxidant (prevents oxidative stress, inhibits the production of free radicals and reduces lipid peroxidation) • Antimicrobial (against *Escherichia coli* LY01 (g-), *Cronobacter sakazakii* (g-) and, *Oenococcus oeni* (g+)) • Anti-diabetic (enhancement of insulin action and C-peptide)	(Cikman et al. 2015) (Shi et al. 2016) (Muthukumaran et al. 2013)
Tartaric acid^a^	00956	0.31	0.32	0.26	• Antimicrobial (against *E. coli*, *Bacillus subtilis* and *Streptococcus suis*)	(Zhihong Gao, 2012)
Vanillate	00484	1.21	2.04	2.20	• Antibacterial (against *Cronobacter* spp) • Anticoagulant (against snake venom)	(Yemis et al. 2011) (Dhananjaya et al. 2006)
Vanillin	12308	0.69	1.18	0.83	• Antioxidant • Anti-inflammatory • Antibacterial (agains *Cronobacter* spp) • Treatment of sickle cell disease	(Makni et al. 2012; Makni et al. 2011) (Yemis et al. 2011) (Abdulmalik et al. 2011)

*ND* Not Detected

^a^Newly identified for expression in rice bran

Amino acids constituted ~28% of the rice bran metabolome from the three rice bran varieties tested. Of the 29 amino acids with medicinal properties in Table 1, 13 had reported antioxidant and/or anti-inflammatory activities, 6 compounds with known antimicrobial properties, 5 that were anti-hypertensive, 4 metabolites with lipid-lowering effects, 3 with cancer chemopreventive actions, one with evidence for prevention of obesity, and one metabolite with hypoglycemic, antidiabetic properties. Multiple metabolites, including taurine and betaine, varied in relative abundance across U.S. varieties, and exhibited more than one mechanism of action with nutritional benefits.

**Fig. 1:**
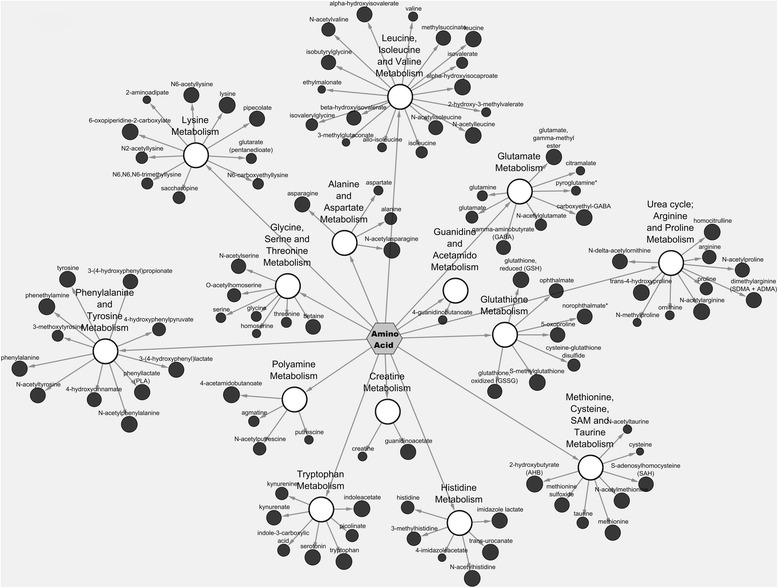
Cytoscape network analysis of rice bran amino acid metabolic pathways. Pathway specific network visualization is shown for Calrose rice bran. Each metabolite is represented as a node (circle), extending from a central sub-metabolic pathway node. The central hexagon represents the super metabolic pathway. Node size corresponds to the Z-score using the relative abundance mean value for all three varieties

Figure 1 is a Cytoscape pathway classification network view specific to amino acids present in Calrose rice bran. The metabolite taurine is presented by a closed black node extending from methionine, cysteine, S-adenosyl- methionine (SAM), & taurine pathway node, which connects to the central, hexagonal amino acid network node. Node size indicates the relative abundance Z-score for the metabolite. The median-scaled relative abundance for each metabolite in Table 1 can be inferred by the size of the node. For example, taurine (methionine, cysteine, S-adenosyl- methionine (SAM), & taurine pathway) has a bigger node size (i.e., bigger Z-score) when compared with betaine (glycine, serine and threonine metabolic pathway).

Cofactors and vitamins constituted ~ 6% of the rice bran metabolome. The 13 metabolites with established medicinal properties are listed in Table 2, with novel identifications of glucarate (median-scaled relative abundance of 0.89, 0.13 for Calrose and Dixiebelle respectively, and non-detectable in Neptune), and threonic acid (median-scaled relative abundance of 36.15, 20.99, and 16.44 for Calrose, Dixiebelle, and Neptune respectively) from rice bran. We found 6 cofactors & vitamins that had antioxidant and/or anti-inflammatory properties, 5 compounds had anti-hypertensive activity, 2 compounds had shown lipid-lowering effects, 4 compounds had evidence for cancer chemopreventive action, and one metabolite was a hypoglycemic agent and antidiabetic.

**Fig. 2:**
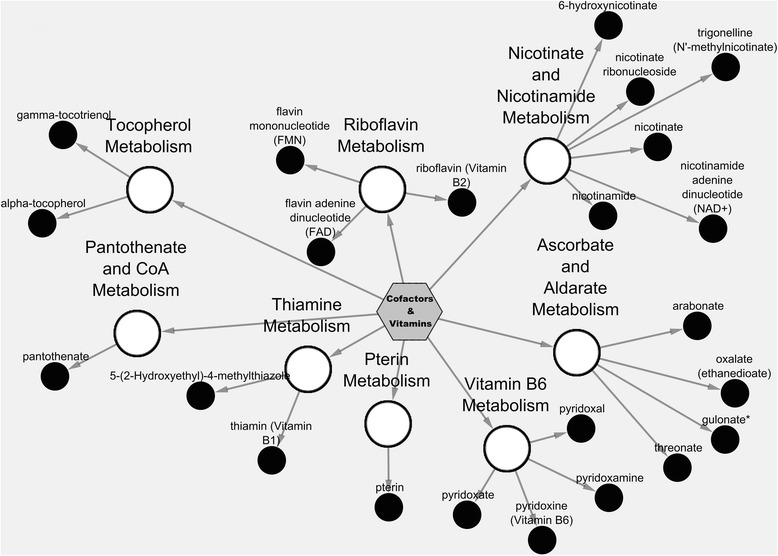
Cytoscape network analysis of rice bran cofactor & vitamin metabolic pathways. Pathway specific network visualization is shown for Calrose rice bran. Each metabolite is represented as a node (circle), extending from a central sub-metabolic pathway node., The central hexagon node represents the super metabolic pathway. Node size corresponds to the Z-score using the relative abundance mean value for all three varieties

Figure 2 shows the Cytoscape pathway classification node network view for cofactors & vitamins metabolic pathways. The micronutrient richness of rice bran is depicted by inclusion of vitamin B6, ascorbate & aldarate, and nicotinate & nicotinamide sub-metabolic pathways with metabolite examples of pyridoxine, threonic acid, and nicotinate for each metabolic pathway, respectively.

**Fig. 3:**
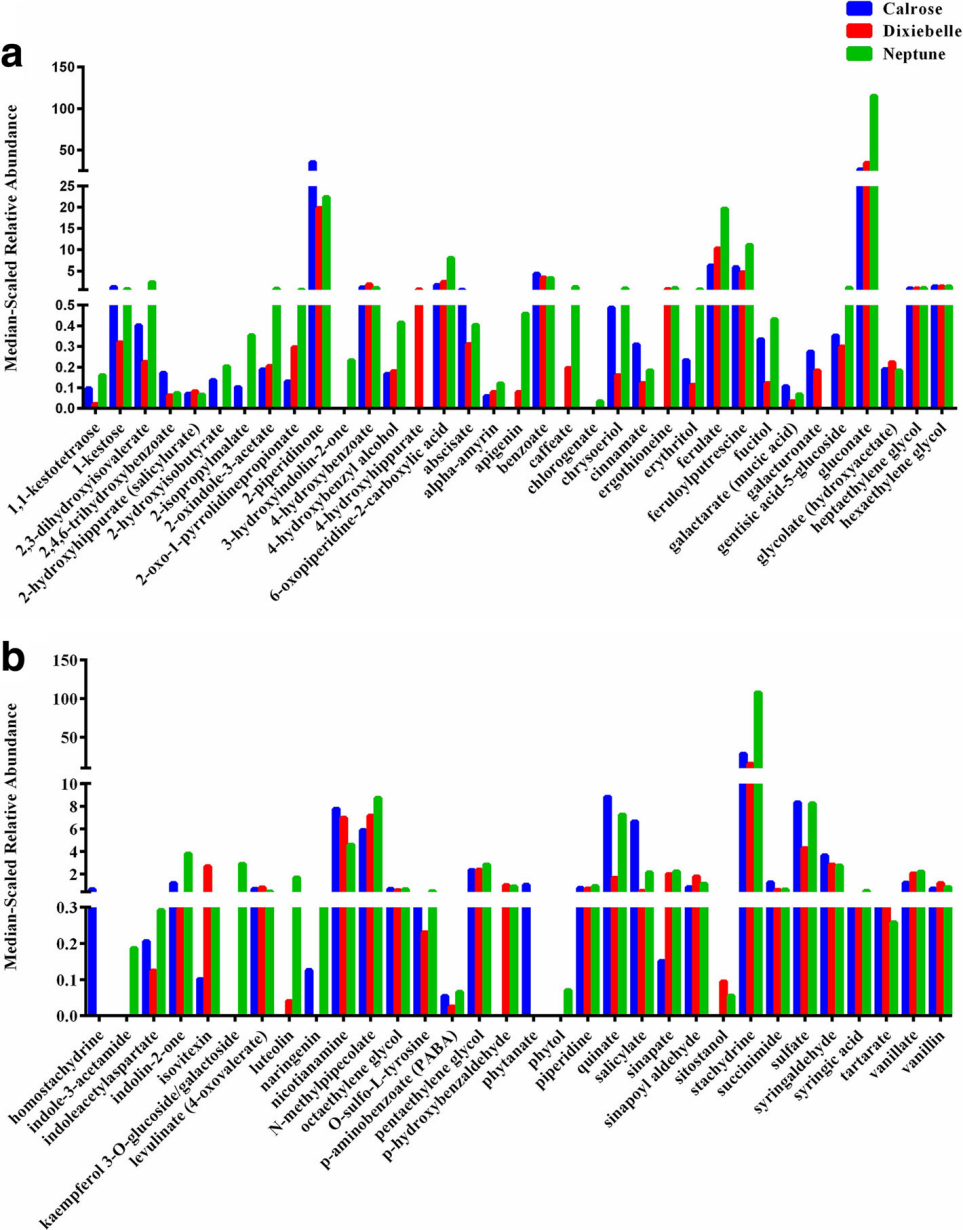
Secondary metabolites detected in rice bran across 3 U.S. varieties. Rice bran metabolites were normalized by median of relative abundance for the entire dataset. **a** shows half of metabolites identified within secondary metabolites metabolic pathways and their difference in median-scaled relative abundance across three varieties. **b** shows another half of identified secondary metabolites and their difference in medin-scaled relative abundance across three varieties

The plant secondary metabolites from rice bran constitute more than 12% of the metabolome. From the 23 rice bran phytochemicals in Table 3 that have known medicinal properties, we identified 16 phytochemicals with antioxidant and/or anti-inflammatory properties, 9 that had antimicrobial effects, 2 components that were anti-hypertensive, 1 with lipid-lowering effects, 4 compounds with evidence of cancer chemoprevention, 1 had scientific reports for utility in obesity prevention, and 4 phytochemicals demonstrated hypoglycemic and anti-diabetic properties. Figure 3 shows all of the secondary metabolites from the rice bran metabolome across three U.S. varieties with respect to their median-scaled relative abundances. Ferulate is an example of a secondary metabolite that has a relative abundance of 6.22, 10.28, and 19.69 in Calrose, Dixiebelle, and Neptune, respectively. Levels of this metabolite are in contrast to tartaric acid, which was 0.31, 0.32, and 0.26. The varied relative abundance detected across metabolites from diverse pathways supports the utility of a metabolome approach.

**Fig. 4:**
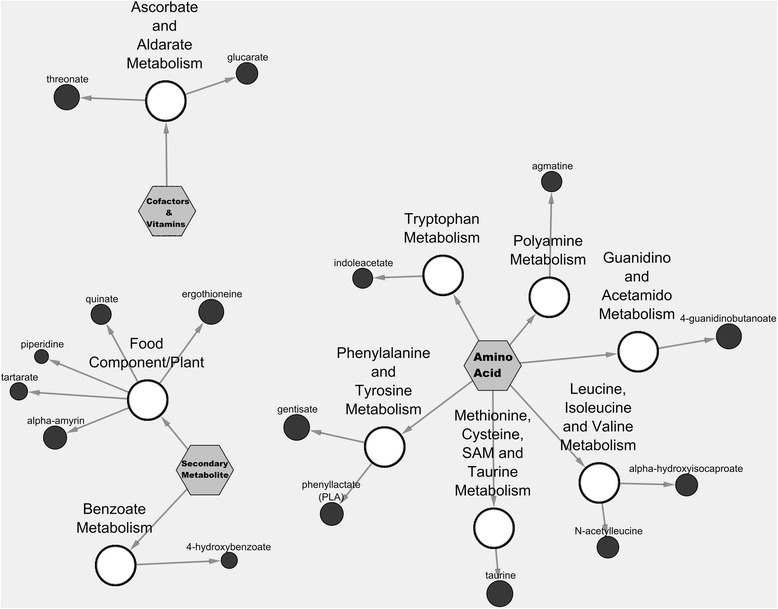
Cytoscape visualisation of 16 newly identified metabolites from the rice bran metabolome. Medicinal and nutritional value for these bioactive compounds are described as deonted by ^1^ in Tables 1, 2 and 3. Pathway specific network visualization is shown for Calrose rice bran. Each metabolite is represented as a node (circle), extending from a central sub-metabolic pathway node. The central hexagon node represents the super metabolic pathway. Node size corresponds to the Z-score using the relative abundance mean value for all three varieties

Figure 4 is the Cytoscape network view of the 16 newly identified metabolites within these metabolic pathways for Calrose (i.e. amino acids, cofactors & vitamins, and secondary metabolites) and their respective sub-metabolic pathways.

### Calrose, Dixiebelle and Neptune have Similar Bran Metabolomes

**Fig. 5:**
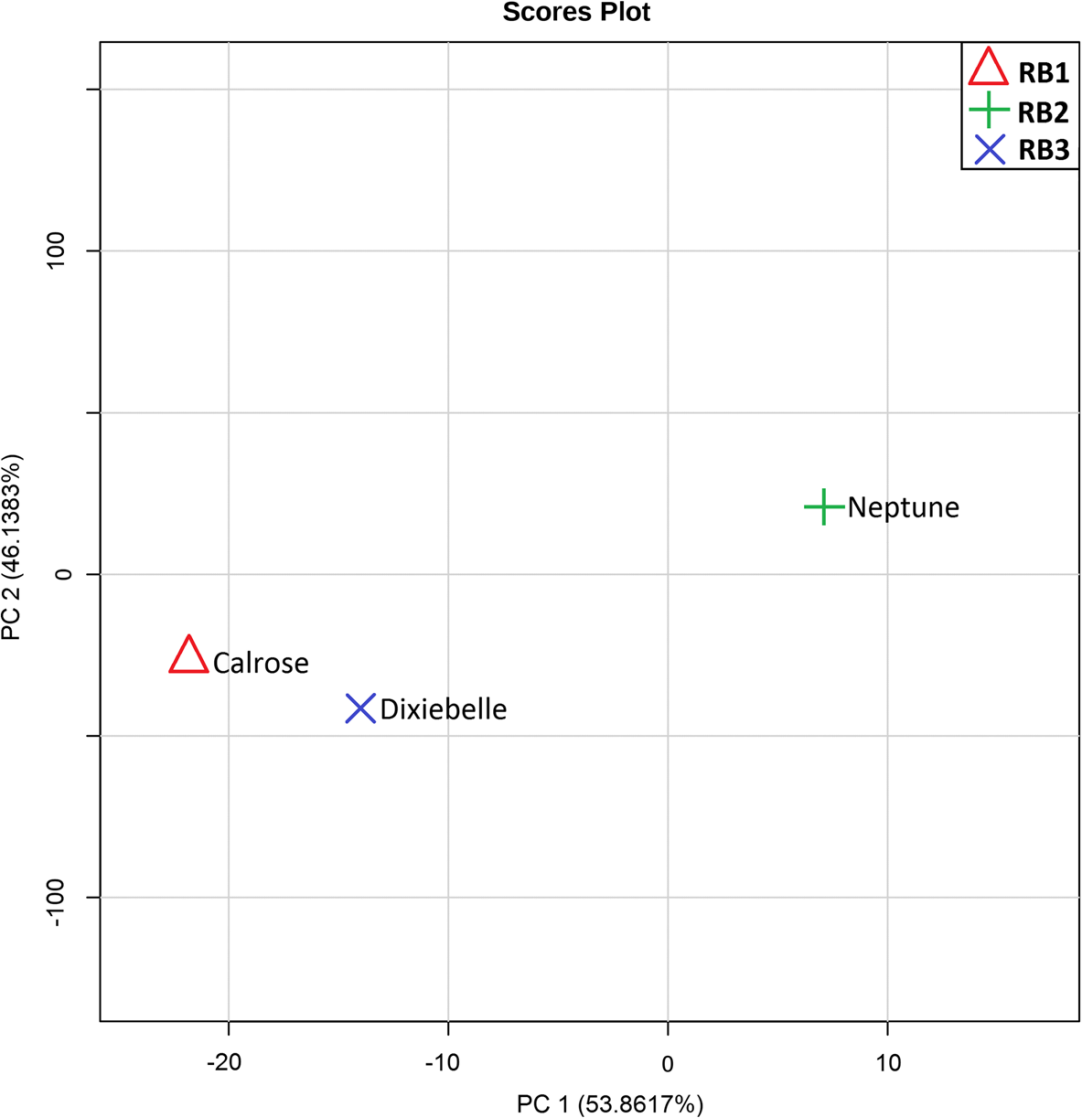
Principal Component Analysis of three U.S. varieties. Principal component analysis (PCA) revealed that the first component described 54% variation herein and between separation of Calrose, Dixiebelle and Neptune rice bran cultivars. The second component describing 46% of the variance did not separate the varieties. No statistical significance was established between the metabolites detected from each of the three U.S rice varieties analyzed

To assess the potential for differences in the relative abundances of small molecules in Calrose, Dixiebelle, and Neptune, we performed a principal component analysis (PCA). Principal component 1 revealed 54% variance among these three rice bran cultivars (Fig. 5), yet this percent variation using PCA did not reach statistical significance, and a lower level of variation is expected between these three U.S varieties when compared to a larger sample set of global varieties with established genome diversity.

## Discussion

The rice bran metabolome analysis herein focused on amino acid, cofactor & vitamin, and secondary metabolite compounds that exhibited medicinal and nutritional properties with an emphasis on chronic and infectious disease control and prevention. The three selected classes of metabolites represented ~46% of total rice bran metabolite profile. Metabolites were described as antioxidative and anti-inflammatory (35 metabolites), antimicrobial (15 metabolites), anti-hypertensive (12 metabolites), cancer chemopreventive (11 compounds). anti-hyperlipidemic (8 metabolites), anti-hyperglycemic (6 compounds), and anti-obesogenic (2 compounds).

Antioxidants represented a broad class of compounds available from many different foodstuffs (Carlsen et al. 2010). Rice bran is a promising candidate for dietary supplementation and nutritional therapy for prevention of chronic and infectious disease via its antioxidant composition. A majority of the rice bran antioxidants (e.g. 4-guanidinobutanoate and taurine from amino acids, tocopherols and tocotrienols from cofactors & vitamins, and ergothioneine and quinate from secondarymetabolites) work through different mechanisms to combat lipid peroxidation, DNA damage, protein modification, and enzyme inactivation caused by free radicals, in particular reactive oxygen species (ROS) (Lü et al. 2010; Nimse and Pal, 2015). Oxidative stress caused by free radicals damages host cells and may initiate early stage development of chronic diseases such as cancer, heart disease, Alzheimer's disease, arthritis, cataracts, diabetes, and kidney disease (Morales-González, 2013). Antioxidants from rice bran can safely interact with and detoxify free radicals to stop the chain of damaging reactions for disease prevention (Iqbal et al. 2005; Jun et al. 2012; Parrado et al. 2006; Parrado et al. 2003). For example, quinate, an antioxidant that is naturally synthesized in plants and microorganisms is now described from rice bran via metabolomics (Fig. 4). It was shown that consumption of 3000 milligrams of quinic acid ammonium chelate per day can regulate activation of NF-kB (nuclear factor kappa-light-chain-enhancer of activated B cells) and enhances DNA repair by increasing serum thiol levels (Pero et al. 2009). Rice bran derived quinate merits further evaluation for similar antioxidant activities.

The antimicrobial activity of dietary rice bran can be attributed to at least 15 metabolites across amino acids and secondary metabolites. Understanding the relative contribution of rice bran compounds and the mechanisms of antimicrobial action could be helpful in combating emerging and existing problems associated with resistance to antibiotics. Hence, treatment strategies using natural food molecules from rice bran may prevent progression of infection and associated symptoms as a sustainable, globally available long-term solution (Cowan, 1999; Kondo et al. 2011; Srivastava et al. 2014). For instance, luteolin is a rice bran flavonoid (shown in Table 3) that reduced the growth of a variety of gram-positive bacteria and yeast (Singh et al. 2016; Srivastava et al. 2014). We, and others, have previously shown that dietary rice bran has antimicrobial activity in animals and on isolated bacterial strains (Goodyear et al. 2015; Irfan A Ghazi et al. 2016; Kim et al. 2014; Kondo et al. 2011; Kumar et al. 2012; Nealon et al. 2017; Yang et al. 2015; Yang et al. 2014). Our study revealed two newly identified rice bran amino acids (out of 15) and three newly identified rice bran secondary metabolites with antimicrobial properties; Phenyllactic acid and α-hydroxyisocaproic acid (leucic acid) from the amino acid metabolic pathway, and 4-hydroxybenzoate, alpha-amyrin, and tartaric acid from the secondary metabolite metabolic pathway. Phenyllactic acid is found in many bacteria as a metabolic byproduct (e.g. *Lactobacillus* spp.) but not previously identified in any plant sources (Valerio et al. 2004). Leucic acid has been identified in fermented foods, including certain cheeses, wines, and soy sauce (Mero et al. 2010). 4-hydroxybenzoate has been previously found in pistachio hulls (Barreca et al. 2016). Alpha-amyrin is found in Carissa carandas (karanda fruit) (Akansha Singh, 2015). Additionally, tartaric acid has been found in Hibiscus sabdariffa flower (Da-Costa-Rocha et al. 2014). Our metabolomics analysis results suggest that nutritional therapy through rice bran's multi-faceted antimicrobial actions merits testing in medical clinical applications to mitigate microbial resistance.

Rice bran merits attention for being of considerably high nutritional value. These metabolome analyses confirm that it is a rich source of proteins, fats, minerals and micronutrients, such as B vitamins and trace elements. For example, at 12 − 15% protein content and with protein digestibility that is comparable to casein, the macro-nutritional value of rice bran which also contains healthy fats and fibers warrants greater pubic health attention (Saunders, 1990; Wang et al. 1999). Rice bran is also a rich source of B-complex vitamins, particularly thiamine and nicotinic acid, riboflavin and vitamin B_6_. A single serving of rice bran (28 grams in accordance to USDA) delivers more than half of the daily nutritional requirements for thiamine, niacin and vitamin B6 (based on a 2,000 calorie reference diet) (SELFNutritionDada; United States Department of Agriculture, 2016). Vitamins cannot be synthesized by the body and must be ingested, as such inadequate intake or subtle deficiencies in vitamins are risk factors for multiple chronic diseases (Fairfield and Fletcher, 2002). Recent evidence showed intake levels of thiamin, niacin, vitamin B_6_, total folate, and alpha-tocopherol was improved in colorectal cancer survivors consuming rice bran and suggests that foods with multiple bioactive components and nutrients can play a pivotal role in the prevention of chronic diseases such as cancer and cardiovascular disease (Borresen et al. 2016; Borresen EC, 2016).

A major strength of the non-targeted metabolomics approach herein was the identification of novel compounds from rice bran with medicinal properties (Fig. 4). The limitations of non-targeted metabolomics in dietary exposure biomarker discovery platforms arise from metabolite concentrations that can vary across cultivars, and inconsistencies in extraction methods or instrument detection limits. Additional limitations for results interpretations from this study involve the limited information for bioavailability of rice bran compounds. The biological properties for rice bran will be dependent on host bioavailability and bioaccessiblity following ingestion, and thus this rice bran food metabolome investigation will assist to identify rice bran exposure biomarkers of intake in people. The variation in gut microbiota composition is another major factor that can influence bioavailability of food metabolites as well as the biological activities (Conlon and Bird, 2015; Krajmalnik-Brown et al. 2012). This is the first non-targeted whole food metabolome study of rice bran with an investigative focus towards the suites of amino acids, cofactors & vitamins, and secondary metabolites. Additional metabolic pathways and chemical classes of metabolites from this analysis (listed in Additional file 1: Table S1) merit continued investigation for medicinal properties and nutritional value.

## Conclusions

This study identified approximately 453 metabolites from the rice bran metabolome, many of which are described herein as cofactors & vitamins, amino acids and secondary metabolites. These metabolic pathways, among others found in rice bran, have shown positive health effects in animals and humans. The wide range of phytochemicals found in rice bran are likely working synergystically to contribute to rice bran’s functional food properties. The ability of rice bran to fight both infectious and chronic diseases may be in part due to synergistic combinations of phytochemicals, and alongside metabolism by the gut microbiota (Borresen et al. 2016; Sheflin et al. 2016; Sheflin et al. 2015). Rice bran biochemical composition merits further investigation for multiple nutritional therapies and medical food applications.

## Methods

### Rice: Milling and Heat Stabilization of Bran

Rice bran from 3 U.S. rice varieties (Calrose, Dixiebelle, and Neptune), representive of rice production in the southeastern U.S. and California, were chosen for this study (Additional file 2: Table S2). Bran was collected and heat stabilized by the United States Department of Agriculture-Agricultural Research Service (USDA-ARS; Stuttgart, AR) as previously reported and utilized in animal and human studies (Borresen EC, 2016; Goodyear et al. 2015; Sheflin et al. 2016; Sheflin et al. 2015).

Rice was milled using a Yamamoto test whitening machine Rice pal VP-31 T grinder and milling system. This laboratory-based instrument has specifications of high accuracy recovery rate (defined as a maximum of 12% bran removal from the whole rice grain). Rice samples were milled at room temperature prior to bran preparation. Testing sieve No. 20 was used to separate bran from broken rice and hulls into a clean container. Once separated, milled bran was heat stabilized at 110 °C for 6 min to prevent rancidity during storage. Rice bran was stored at −20 °C until further processing for metabolite analysis.

### Rice Bran Extraction

Metabolomics analysis was performed by Metabolon Inc. (Durham, NC). Prior to the first step of extraction, several recovery standards (also called quality control standards) were added into the samples for quality control purposes. Quality control standards, that were carefully chosen not to interfere with the measurement of endogenous compounds, were spiked into every analyzed sample to allow instrument performance monitoring and aid chromatographic alignment. The purpose of adding the standards was to assess variability and verify performance of extraction and instrumentation. Values for instrument and process variability met Metabolon’s acceptance criteria. Rice bran samples were mixed with 80% ice-cold methanol under vigorous shaking for 2 min (Glen Mills GenoGrinder 2000) and then were centrifuged to precipitate protein, and free small molecules bound to other macromolecules. The supernatant fraction, i.e., rice bran extract (RBE), was used for further analysis and was divided into four portions: three portions for different mode of analysis by ultra-performance liquid chromatography-tandem mass spectrometry (UPLC-MS/MS) (i.e., one portion for analysis with positive ion mode electrospray ionization, one for negative ion mode electrospray ionization, and one portion for analysis by UPLC-MS/MS polar platform (negative ionization), and one portion for analysis by gas chromatography–mass spectrometry (GC-MS)). Samples were placed briefly on a TurboVap® (Zymark) concentration evaporator to remove the organic solvent. For UPLC, the samples were stored overnight under nitrogen before preparation for analysis. For GC, each sample was dried under vacuum overnight before preparation for analysis.

### Ultra-Performance Liquid Chromatography-Tandem Mass Spectroscopy (UPLC-MS/MS)

The UPLC-MS/MS was performed for a non-targeted metabolomics analysis based on a Waters ACQUITY ultra-performance liquid chromatography (UPLC) and a Thermo Scientific Q-Exactive high resolution/accurate mass spectrometer interfaced with a heated electrospray ionization (HESI-II) source and Orbitrap mass analyzer operated at 35,000 mass resolution. The dried RBE was re-suspended in acidic or basic UPLC-compatible solvents. Each sample contained 8 injection quality control standards at fixed concentrations to ensure injection and chromatographic consistency. These standards, in concert with experimental samples, generated a pooled matrix served as technical replicates throughout the data set across all samples and varieties. The acidic solution was analyzed using acidic positive ion optimized conditions, and the basic solution was analyzed using basic negative ion optimized conditions under two identical separate dedicated columns independently (Waters UPLC BEH C18-2.1x100 mm, 1.7 μm). For acidic conditions, RBE was eluted at 350 μl/min from a C18 column using (A) 0.1% formic acid in water and (B) 0.1% formic acid in methanol (0% B to 70% B in 4 min, 70-98% B in 0.5 min, 98% B for 0.9 min). Likewise, the basic extracts were eluted from C18 using ammonium bicarbonate instead of formic acid. Another portion of RBE was also analyzed through negative ionization using bicarbonate used (A) 6.5 mM ammonium bicarbonate in water, pH 8, and (B) 6.5 mM ammonium bicarbonate in 95/5 methanol/water (same gradient profile as above) at 350 μL/min, followed by elution from a hydrophilic interaction liquid chromatography (HILIC) column (Waters UPLC BEH Amide 2.1x150 mm, 1.7 μm). 10 mM ammonium formate were gradient eluted at 500 μL/min using (A) 10 mM ammonium formate in water and (B) 10 mM ammonium formate in acetonitrile (5% B to 50% B in 3.5 min, 50% B to 95% B in 2 min and 95% B for 1 min). The MS analysis alternated between MS and data-dependent MS2 scans using dynamic exclusion, and the scan range was from 80–1000 m/z (Brown et al. 2016).

### Gas Chromatography-Mass Spectroscopy (GC-MS)

The RBEs assigned for GC-MS analysis were dried under vacuum overnight (18 h). They were further derivatized with bistrimethyl-silyltrifluoroacetamide under dried nitrogen. Derivatized RBEs were separated on a 5% diphenyl/95% dimethyl polysiloxane fused silica column (20 m x 0.18 mm ID; 0.18 um film thickness) with an appropriate carrier gas. Samples were analyzed on a Thermo-Finnigan Trace DSQ™ fast-scanning single-quadrupole mass spectrometer using electron impact ionization (EI) and operated at unit mass resolving power. The scan range was from 50–750 m/z (Brown et al. 2016).

### Metabolite Data Extraction and Compound Identification

Raw instrument data was extracted and then processed through Metabolon in-house developed peak detection and integration software (quantitation is based on area under the curve from MS data). This software uses standard industry approaches for MS peak detection, including using minimum height, signal-to-noise, width and area criteria. These systems are built on a web-service platform utilizing Microsoft’s .NET technologies, which run on high-performance application servers and fiber-channel storage arrays in clusters to provide active failover and load-balancing. Compounds were identified by comparison to library entries of purified standards or recurrent unknown entities. Metabolon maintains a library based on authenticated standards that contains the retention time/index (RI), mass to charge ratio (m/z), and chromatographic data (including MS/MS spectral data) on all molecules present in the library. Biochemical identifications were based on three criteria: the experimentally detected signature matching the accurate mass of the authentic standard within 8 ppm, i.e. match to the NIST (National Institute of Standards and Technology) library within +/− 0.005 atomic mass units), retention index match within a defined window (approximately 5 s), and the Tandem mass spectrometry (MS/MS) forward and reverse scores between the experimental data and high quality standards. The MS/MS scores are based on a comparison of the ions present in the experimental spectrum to the ions present in the library spectrum. There are more than 3300 commercially purified standard compounds that have been registered in Metabolon Laboratory Information Management System for distribution to both the LC-MS and GC-MS platforms for determination of their analytical characteristics (Evans AM, 2014; Evans et al. 2009).

### Statistical Analysis

A data normalization step was performed to correct variation between instrument inter-day tuning differences. Each compound was corrected in run-day blocks by registering the medians to equal 1.00 and normalizing each data point proportionally. The relative abundance of each metabolite was also normalized by median of the metabolite across the entire dataset (i.e. median-scaled). Median-scaled relative abundance is calculated as: raw abundance of a metabolite/median raw abundance of that metabolite across the entire dataset. Median-scaled relative abundance Z-score was further used as a basis for metabolic pathway network visualization. Z-scores are expressed as standard deviations from the mean and were calculated using the following formula: z = (x- μ)/σ where “x” is median-scaled relative abundance of the metabolite, “μ” is mean of median-scaled relative abundance for the metabolite across three rice bran varieties, and “σ” is the median-scaled relative abundance standard deviation of same metabolite across three varieties. Metabolic pathway and the graphical presentation of metabolite interaction network were composed with Cytoscape version 3.4.0.

## Additional files

Additional file 1: Table S1. Number of metabolites identified in rice bran metabolic pathways. (DOCX 12 kb)

Additional file 2: Table S2. Rice plant phenotypic characteristics. (DOCX 14 kb)
