# Supplementary material for: Rice Bran Metabolome Contains Amino Acids, Vitamins & Cofactors, and Phytochemicals with Medicinal and Nutritional Properties
Source: Rice (N Y). 2017 Jun 2;10:24. doi: 10.1186/s12284-017-0157-2 (PMC5453916; doi:10.1186/s12284-017-0157-2)
Supplement: Supplementary file 1 — Number of metabolites identified in rice bran metabolic pathways. (DOCX 12 kb) [file 12284_2017_157_MOESM1_ESM.docx]

| Table S1. Number of metabolites identified in rice bran metabolic pathways | | | |
| --- | --- | --- | --- |
| Metabolic pathways | Calrose | Dixiebelle | Neptune |
| *Amino acid | 120 | 113 | 126 |
| Carbohydrate | 35 | 34 | 32 |
| *Cofactors & vitamins | 28 | 28 | 25 |
| Energy | 11 | 11 | 11 |
| Lipid | 137 | 135 | 128 |
| Nucleotide | 37 | 35 | 40 |
| Peptide | 25 | 25 | 28 |
| *Secondary metabolites | 47 | 50 | 55 |
| Xenobiotics | 8 | 7 | 8 |
| Total number of detected metabolite | 448 | 438 | 453 |
