# Supplementary material for: Rice Bran Metabolome Contains Amino Acids, Vitamins & Cofactors, and Phytochemicals with Medicinal and Nutritional Properties
Source: Rice (N Y). 2017 Jun 2;10:24. doi: 10.1186/s12284-017-0157-2 (PMC5453916; doi:10.1186/s12284-017-0157-2)
Supplement: Supplementary file 2 — Rice plant phenotypic characteristics. (DOCX 14 kb) [file 12284_2017_157_MOESM2_ESM.docx]

| **Table S2. Rice plant phenotypic characteristics** | | | | | | | | | |
| --- | --- | --- | --- | --- | --- | --- | --- | --- | --- |
| **Rice Bran Variety** | **Year of Production** | **Location Produced** | **Bran Color** | **Sub population** | **Grain Type** | **Seed Sources** | **Amylose Content** | **Gelatinization Temperature** | **Yield** |
| **Calrose** | 2012 | Nelson, CA | Brown | Temperate japonica | Medium Grain | Commercial Production | Low | Low | High |
| **Neptune** | 2009 | Crowley, LA | Brown | Tropical japonic | Medium Grain | 09/LSU/Headrow | Low | Low | high |
| **Dixiebelle** | 2009 | Beaumont, TX | Brown | Tropical japonic | Long Grain | 2009 USDA Headrow | High | Intermediate | Moderate |
